# Draft Genome Sequence of Streptococcus halitosis sp. nov., Isolated from the Dorsal Surface of the Tongue of a Patient with Halitosis

**DOI:** 10.1128/MRA.01704-18

**Published:** 2019-01-24

**Authors:** George Tetz, Daria Vikina, Stuart Brown, Paul Zappile, Igor Dolgalev, Aristotelis Tsirigos, Adriana Heguy, Victor Tetz

**Affiliations:** a Human Microbiology Institute, New York, New York, USA; b Tetz Laboratories, New York, New York, USA; c Applied Bioinformatics Laboratories, New York University Medical Center, New York, New York, USA; d Department of Pathology, New York University School of Medicine, New York, New York, USA; e Laura and Isaac Perlmutter Cancer Center, New York University School of Medicine, New York, New York, USA; f Genome Technology Center, Division of Advanced Research Technologies, NYU School of Medicine, New York, New York, USA; University of Southern California

## Abstract

Here, we report the draft genome of Streptococcus halitosis sp. nov. strain VT-4, a novel bacterium isolated from the dorsal part of the tongue of a patient with halitosis. The genome comprised 1,880,608 bp with a GC content of 41.0%. There were 1,782 predicted protein-coding genes, including those associated with virulence and antibiotic resistance.

## Abstract (precis)

Here, we report the draft genome of Streptococcus halitosis sp. nov. strain VT-4, a novel bacterium isolated from the dorsal part of the tongue of a patient with halitosis. The genome comprised 1,880,608 bp with a GC content of 41.0%.

## ANNOUNCEMENT

Streptococcus is a genus of nonmotile, Gram-positive, coccus-shaped bacteria. These bacteria cause a variety of human pathologies, including some associated with the oral cavity, such as dental caries, tartar, and gingivitis (1, 2). However, none of the Streptococcus species are associated with halitosis that is characterized with an increase in the number of volatile sulfur compounds (VSC), particularly due to H_2_S-producing microorganisms on the tongue (3).

Streptococcus halitosis sp. nov. strain VT-4 was isolated from the dorsal part of the tongue of a patient with halitosis using an original workflow as previously described (4–6). For genomic DNA isolation, a single colony grown on Columbia agar at 37°C. Genomic DNA was extracted using a genomic DNA purification kit (QIAamp). The 16S rRNA gene was amplified with the universal bacterial primers 27F and 1492R and assembled using SeqMan v7 software (7, 8). The 16S rRNA gene of Streptococcus halitosis VT-4 possesses 99% sequence identity with S. oralis and 98% sequence identity with S. infantis, S. mitis, and S. pneumoniae (9).

Paired-end libraries (300-bp length) were prepared using a TruSeq DNA sample prep kit and then sequenced using a HiSeq 2500 instrument (Illumina, USA). Samples underwent library preparation and sequencing according to the manufacturer’s instructions.

Raw sequence reads were cleaned with Trimmomatic (v0.38) to remove Illumina adapters and trimmed within a sliding 4-base window, cutting when the average quality per base dropped below 15 (10). Contaminating human sequences were removed by alignment to the human reference genome (GRCH38) with Bowtie 2 (v2.3.4.2), collecting only unaligned read pairs (11). A draft genome was assembled using SPAdes v3.7.1 with default parameters (12). Contigs with an average kmer coverage of less than 10-fold were deleted.

The assembled 18 contigs had an average kmer coverage of 130-fold, with a total length of 1,880,608 bp, a GC content of 41.0%, and an *N*_50_ value of 1,331,771 bp. The assembled sequences were annotated using the NCBI Prokaryotic Genome Annotation Pipeline (13). The genome of S. halitosis VT-4 harbored 49 tRNA genes, 4 rRNAs, and 3 noncoding RNA (ncRNA) operons, and it had 1,782 protein-coding sequences.

We revealed multidrug resistance transporters of the ATP-binding cassette, multidrug and toxic compound extrusion, and major facilitator superfamily families. Furthermore, resistance determinants, such as an aminoglycoside phosphotransferase and a BlaEC family class C beta-lactamase, were found. Virulence factors, including hemolysin III, metalloproteases, serine proteases, endopeptidases, deoxyribonucleases, ribonucleases, and adhesins, were identified in the genome.

We discovered the presence of cystathionine beta-lyase and l-lactate dehydrogenase genes, associated with the formation of hydrogen sulfide (H_2_S), known as a predominant VSC associated with halitosis (3, 14, 15).

An *in silico* DNA-DNA hybridization (dDDH) was carried out using the Genome-to-Genome Distance Calculator (16–18). The results revealed that the S. halitosis VT-4 genome was distinct from the genomes of representative strains of related species (i.e., S. oralis, S. infantis, S. mitis, and S. pneumoniae, with similarity values of 60.00, 25.80, 25.80, and 31.40%, respectively) and were below the 70% cutoff for dDDH (16, 17).

Further studies of S. halitosis VT-4 and bacteriophages associated with this bacterium will enhance our understanding of its implications in human pathologies of the oral cavity (19, 20).

### Data availability.

The complete genome sequence of Streptococcus halitosis sp. nov. strain VT-4 has been deposited in the NCBI database under accession number QEMY00000000. Run data are available from the Sequence Read Archive (SRA) under the accession number SRR8172788.
